# Supplementary material for: Lysine Methyltransferase SMYD1 Regulates Myogenesis via skNAC Methylation
Source: Cells. 2023 Jun 22;12(13):1695. doi: 10.3390/cells12131695 (PMC10340688; doi:10.3390/cells12131695)
Supplement: Supplementary file 1 [file cells-12-01695-s001.zip › cells-2408167-supplementary.pdf]

SUPPLEMENTARY FIGURES

S-Figure 1

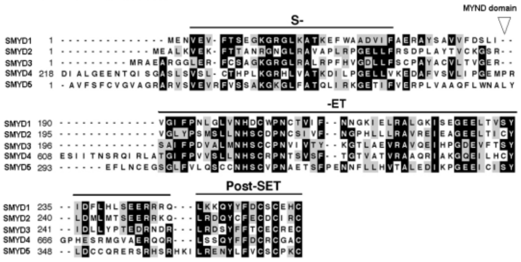

Figure S1. Comparison of the split SET domains and the post-SET domains present in SMYD1, Smyd2, Smyd3, Smyd4 and Smyd5. Conserved residues are indicated by black shading, similar residues are indicated by gray. The location of the MYND domain is indicated by an open arrowhead.

S-Figure 2

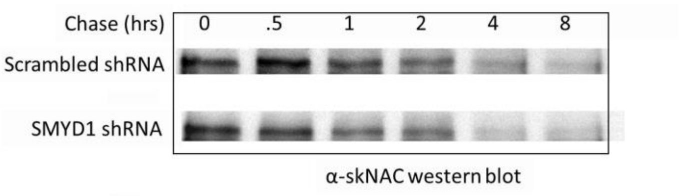

Figure S2. Loss of SMYD1 has no effect on the stability of skNAC. C2C12 cells were stably transduced with either SMYD1 shRNA or a scrambled sh-RNA, biosynthetically labeled with 3H-SAM, and chased over a time course of 0 to 8 hr, and skNAC was

immunoprecipitated, then analyzed by SDS-PAGE western blot. No significant differences were observed between SMYD1-deficient and scrambled control.

S-Figure 3

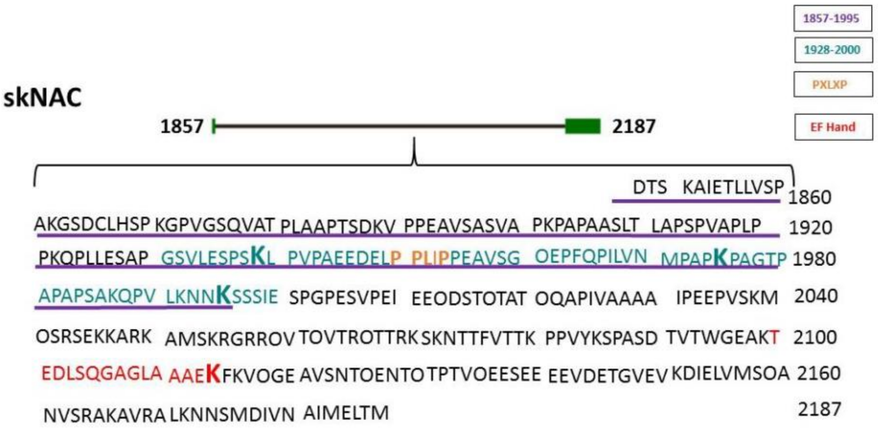

Figure S3. **Schematic of skNAC carboxyl terminal residues 1857-2187** with all the lysine residues mutated in skNAC (1928-2100) (green color and capitalized) and the PXLXP domain (orange color).

S-Figure 4

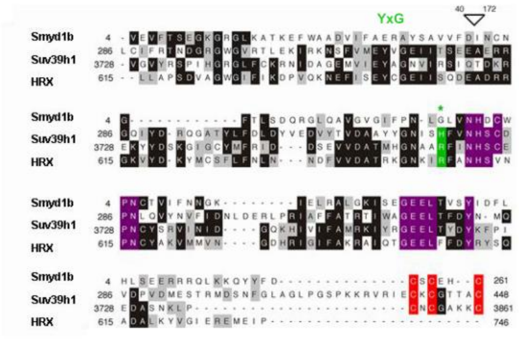

Figure S4. Comparison of the SET domain and the post-SET domain present in Smyd1 and other HMTases. Conserved residues are indicated by black shading; similar residues are indicated

by gray and residues necessary for HMTase activity are indicated by purple or red. The prominent difference between Smyd1 and other HMTases at residues critical to HMTase activity are shown in Green. The location of the MYND domain is indicated by an open arrowhead.

S-Figure 5

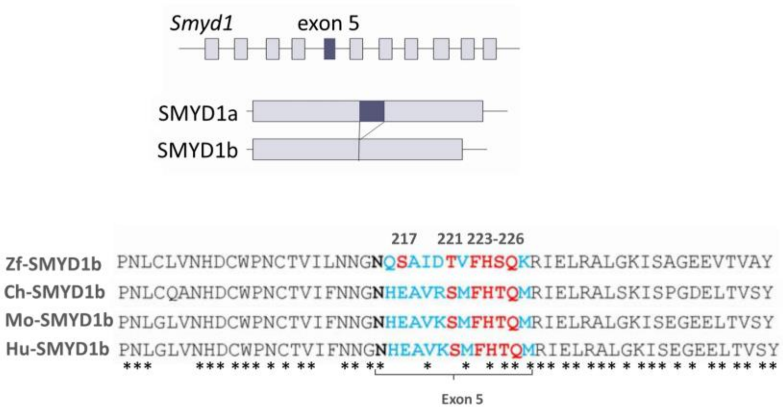

Figure S5. Conservation and features of alternatively spliced exon 7 of SMYD1a. Upper panel: SMYD1a and SMYD1b differ by a 13 amino acid insertion within the SET domain due to alternative inclusion of Exon 5. Lower panel: Sequence conservation of Exon 5 among vertebrate orthologues. Potential S/T phosphorylation sites are conserved at amino acid positions 217 and 221; a conserved tetramer (FHSQ) is conserved near the C-terminal end of Exon 3 at residues 223-226. Mm, *mus musculis*; Hs, *homo sapiens*; Gg, *gallus gallus*; Zf, *zerebrafish*; Ch, *cephalochordate*. *, amino acid identities.
